# Understanding the Functional Activity of Polyphenols Using Omics-Based Approaches

**DOI:** 10.3390/nu13113953

**Published:** 2021-11-05

**Authors:** Wenjin Si, Yangdong Zhang, Xiang Li, Yufeng Du, Qingbiao Xu

**Affiliations:** 1 College of Animal Sciences and Technology, Huazhong Agricultural University, Wuhan 430070, China; 2021302120065@webmail.hzau.edu.cn (W.S.); xxianglli@mail.hzau.edu.cn (X.L.); duyufeng@webmail.hzau.edu.cn (Y.D.); 2 Shennongjia Science & Technology Innovation Center, Huazhong Agricultural University, Wuhan 430070, China; 3 State Key Laboratory of Animal Nutrition, Institute of Animal Science, Chinese Academy of Agricultural Sciences, Beijing 100193, China; zhangyangdong@caas.cn

**Keywords:** polyphenols, foodomics, functional activity, gut microbiota, multi-omics

## Abstract

Plant polyphenols are the main category of natural active substances, and are distributed widely in vegetables, fruits, and plant-based processed foods. Polyphenols have a beneficial performance in preventing diseases and maintaining body health. However, its action mechanism has not been well understood. Foodomics is a novel method to sequence and widely used in nutrition, combining genomics, proteomics, transcriptomics, microbiome, and metabolomics. Based on multi-omics technologies, foodomics provides abundant data to study functional activities of polyphenols. In this paper, physiological functions of various polyphenols based on foodomics and microbiome was discussed, especially the anti-inflammatory and anti-tumor activities and gut microbe regulation. In conclusion, omics (including microbiomics) is a useful approach to explore the bioactive activities of polyphenols in the nutrition and health of human and animals.

## 1. Introduction

Plant polyphenol is a kind of secondary metabolites in plants and can enhance plant resistance and stress from disease and external environment. Generally, polyphenols are widely presented in the plant tissues (e.g., stems, roots, leaves, flowers, and fruits) as the most common plant active substances in nature [1]. Plant polyphenols have various biological activities, including anti-tumor, anti-cardiocerebrovascular, anti-oxidative, anti-aging, anti-inflammatory, and anti-viral activities [2,3,4,5,6,7]. An increasing number of studies have shown that plant polyphenols play a positive role in improving animal growth performance and maintaining gastrointestinal health as antibiotics [8,9,10,11]. There is evidence that polyphenols (1000 mg/kg) can improve the intestinal morphology of yellow feather broilers, increase the body’s antioxidant capacity, and improve the quality of chicken [12]. Moreover, DSS-induced colitis can be prevented and treated by honey polyphenols through regulating gut microbiota [13,14]. Therefore, polyphenols have a great potential to be a green antibiotic substitute with a beneficial effect in human and animals.

Currently, the studies on polyphenols from plant extract are mainly focused on their composition, health benefits, and metabolism [15,16]. The potential health benefits of plant polyphenols were confirmed by numbers of previous reports [17,18,19]. In a previous study, as the intake of green tea increased (≥2 times/day), the incidence of chronic obstructive pulmonary disease dropped from 14.1% to 5.9%, and it was inferred that consumption of green tea polyphenol was associated with a reduction in the risk of certain disease [20]. Another study showed that resveratrol (10 and 20 mg/kg) reduced oxidative stress and inflammation. Therefore, resveratrol may be a potential therapeutic strategy for the treatment and prevention of diabetic encephalopathy [21]. Similarly, supplementing the diet with concentrated red grape juice (100 mL/day) can reduce the plasma concentration of inflammatory biomarkers and oxidized low-density lipoproteins, and may be beneficial to reducing cardiovascular disease risk [22]. However, their metabolic pathways and regulating mechanisms have not been fully clarified. Fortunately, foodomics technology provides a novel option for investigating the functional mechanism of polyphenols [23,24]. Foodomics mainly consist of genomics, transcriptomics, proteomics, metabolomics, and microbiome, and foodomics has been widely used to study how to maintain gut health and normal metabolisms by regulating gut microbiota with the influence of polyphenols [25].

Recent years, mono-omics was used widely in polyphenol research. However, owing to its own limitations, mono-omics cannot fully and systematically explain the action mechanism of polyphenols. Foodomics has the advantages of diversity, relevance, and systematicity in multi-omics. For exploring and developing new insights in the molecular mechanisms of novel biomarkers, foodomics is used to analyze the biological activity of the effective compounds in food. Therefore, foodomics is an inevitable trend in the development of polyphenol study. In this review, the strategies of foodomics were discussed in investigating the activity of polyphenols and the action mechanisms and application of polyphenols.

## 2. Classification, Source and Function of Polyphenols

More than 8000 phenolic substances are commonly distributed in fruits, vegetables, tea, coffee, cocoa, beans, and grains (Table 1) [26]. Polyphenols have complex structures and can be divided into phenolic acids, lignans, stilbene, tannins, and flavonoids (e.g., isoflavones and anthocyanins). Polyphenols derived from various sources have many beneficial and specific therapeutic properties (Table 1). Phenolic acid has an extensive physiological activity, including anti-oxidation, scavenging free radicals, anti-ultraviolet radiation, and antibacterial and antiviral effects. Stilbene resveratrol has a preventive effect on atherosclerosis and cancer [27]. Stilbene and flavonoids can be used to prevent and treat cardiovascular and cerebrovascular diseases [28,29,30]. As the most common phytoestrogens, lignans are famous for its high anti-oxidant activity and inhibiting lipid peroxidation [31,32]. Lignans can also bind to estrogen receptors and interfere with cancer-promoting effects; therefore, it has a preventive effect on breast and colon cancer. As a kind of polyphenols, tannin can exert various activities, such as anti-oxidative, anti-microbial, anti-cancer, anti-hypertensive, and anti-inflammatory effects [33,34]. However, complexes can be formed by polyphenols with starch, protein, and enzymes; therefore, they are considered as anti-nutrients. Due to their carcinogenic and anti-nutritional effects, it is harmful for human and animal to have too many tannins [35].

Due to their extensive biological activities, plant polyphenols have become a study hotspot in the field of human nutrition and health. Similarly, polyphenols also have various positive effects on livestock and poultry. Plant polyphenol extracts and polyphenol monomer compounds can effectively improve animal intestinal microenvironment with various functional activities, such as immune regulation, bacteriostasis, anti-oxidation, and microbiota regulation [36,37].

## 3. Foodomics Applied in the Study of Polyphenols

**Figure 1:**
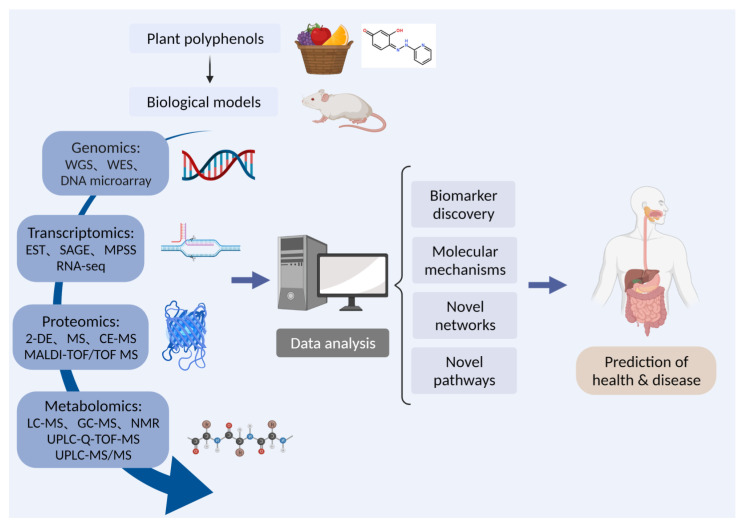
The strategy of foodomics to study the bioactivities of polyphenols. 2-DE: two-dimensional gel electrophoresis; CE-MS: capillary electrophoresis mass spectrometry; EST: expression sequence tags technology; GC: gas chromatography; LC: liquid chromatograph; MPSS: massively parallel signature sequencing; MALDI-TOF/TOF: matrix-assisted laser desorption ionization time-of-flight/time-of-flight; MS: mass spectrometry; NMR: nuclear magnetic resonance; RNA-seq: RNA sequencing; SAGE: serial analysis of gene expression; UPLC-Q-TOF: ultra-performance liquid chromatography to quadrupole time-of-flight; WES: whole exome sequencing; WGS: whole genome sequencing.

As shown in Figure 1, foodomics is a collection of genomics, transcriptomics, proteomics, and metabolomics, and can be used to study polyphenols from multiple angles. The data obtained from various omics will be integrated to explore the molecular mechanism and novel pathways of plant polyphenols to predict and treat diseases of human and animals.

### 3.1. Genomics

Genomics, containing genome sequencing and analysis, is used to explore the interrelationships and impacts on organisms by characterizing collectively and quantifying all genes of tissue [69]. Up to now, genome sequencing technology has developed to the third or even fourth generation with various nonnegligible advantages, such as high throughput, fast speed, and high accuracy. Besides, the sequencing technology of genomics mainly includes whole-exome sequencing, whole-genome sequencing, and DNA microarray technology. Genomics has created a precedent in the era of omics and is the foundation of foodomics. It plays a vital role in exploring the bioactivity of polyphenols via sequencing and explaining the underlying mechanism at the DNA level. To evaluate the anti-inflammatory function and targeting genes of polyphenols, macrophages were treated using polyphenols and their gene expression profile was analyzed using DNA microarrays. It was found that bilberry polyphenols can decrease the high expression level of inflammatory genes caused by lipopolysaccharide [70]. Using DNA microarray technology, polyphenols from oolong tea was found to exert anti-inflammatory effects by regulating molecular networks, such as cytokines, interleukins and interferons [71].

Genomics technology can facilitate to understand the action mechanism of polyphenols and discover novel natural polyphenols. Many genomics technologies are the effective tools for identifying polyphenol genes, including candidate gene methods, quantitative trait locus (QTL) detection, and genome-wide association studies (GWAS). As the name implies, QTL refers to the position of genes that control quantitative traits in the genome. These QTLs were detected in apples for the first time and represent a novel step in studying the biosynthesis mechanism of proanthocyanidins and the biosynthesis of phenolic compounds. It was used in the measurement of main phenolic compounds in sensory properties and main polymerization degree of proanthocyanidins in cider [72]. GWAS involve the whole-genome resequencing (WGS) of each individual in a population with rich genetic diversity. The phenotypic data of the target trait is integrated for whole-genome association analysis, which can quickly obtain chromosome segment or gene locus that affects the target trait. In a previous study, WGS data were used to perform GWAS analysis on 10 polyphenol components, unearthing key QTLs, screening specific germplasm, and discovering excellent genes. This analysis greatly accelerated the breeding process of polyphenol-rich varieties [73]. The WGS combined with bioinformatics analysis was used and discovered the five-membered angle ring polyphenols with novel structure [74]. Therefore, it can be concluded that the combination of GWAS and other genetic technologies represents the trend of the future research on polyphenols.

### 3.2. Transcriptomics

**Figure 2:**
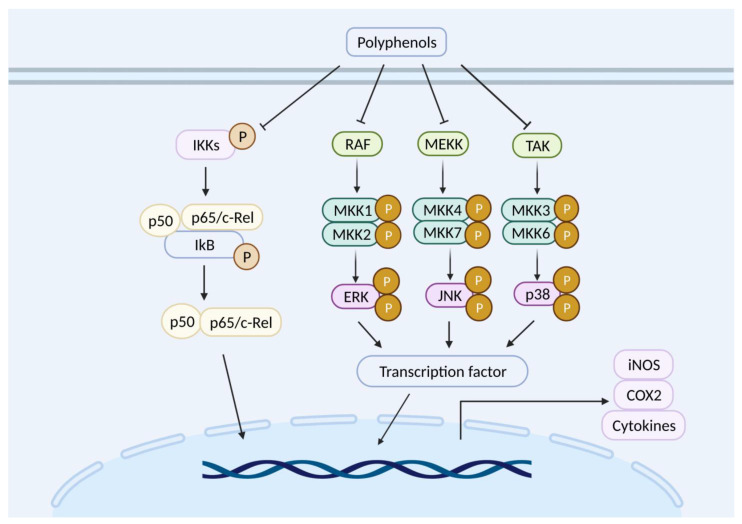
The potential anti-inflammatory mechanism of polyphenols. COX2: cyclooxygenase-II; ERK: extra-cellular signal regulated kinases; IKKs: IkB-kinase; iNOS: inducible nitric oxide synthase; JNK: c-Jun amino-terminal kinases; MEKK: mitogen-activated protein kinase kinase kinase; MKK: mitogen-activated protein kinase kinase; NF-kB: nuclear factor kappa-light-chain-enhancer of activated B cells; RAF: Raf kinases; TAK: Tak kinases.

Transcriptome is a total number of RNA transcribed from a specific cell or tissue in a certain functional state. Transcriptomics is used to investigate the transcription conditions and transcriptional regulation rules from the overall level. The definition of time and space is the difference between the transcriptome and genome. Polyphenols can change the expression of genes in the inflammation related signal pathway, regulating the NF-κB signal pathways by inhibiting the activity of IKKs, preventing p50 and p65 from entering the nucleus, and enhancing the expression of a series of inflammatory cytokines (e.g., iNOS, cyclooxygenase-2 (COX-2), and cytokines) [75,76] (Figure 2). It can also regulate monophosphate-activated protein kinase (MAPK) pathway by alleviating the activity of MAPKKKs and prevent a series of transcription factors entering the nucleus to express a series of inflammatory cytokines [77]. Baicalein can decrease COX-2 expression via regulating MAPK signal pathway [78]. Similarly, quercetin can also decrease COX-2 expression through regulating the NF-κB signal pathway [79]. COX-2 is a kind of cyclooxygenase with very low activity in normal cells. However, when the cells are stimulated by inflammation, the expression level of COX-2 can be increased to dozens of times, causing inflammation and tissue damage. Secondly, polyphenols display an anti-oxidant function through regulating transcription factor level and activating signal channels to enhance the expression of anti-oxidant proteins. Quercetin can up-regulate the transcription level of nuclear factor erythroid-2-related factor (Nrf2), post-transcriptional level, and inhibit the post-transcriptional expression of Keap1, resulting in the enhancement of the expression of anti-oxidant proteins and detoxification enzymes [80]. Similarly, fisetin and mustard extracts can up-regulate the expression of reducing coenzyme II-quinone oxidoreductase through transcriptional activation of the Nrf2-ARE anti-oxidant pathway [81,82].

At present, transcriptomics technology mainly consists of two types: microarray based on hybridization, and transcriptome sequencing technology based on sequencing technology, which includes expression sequence tags technology, RNA sequencing, gene expression analysis, and signature sequencing. cDNA microarray-based transcriptome technology was used to illustrate the important role of microRNAs in resveratrol-mediated colon cancer associated with colitis. In detail, resveratrol can mediate anti-inflammatory properties and inhibit gut tumorigenesis via miRNA regulation [83]. In addition, the full-length transcriptome sequencing technology was combined to reveal the expression of olive polyphenol anabolism-related genes, which facilitates to understand the polyphenol biosynthesis pathway in olives [84]. Due to the characteristics of high anti-oxidant performance, good thermal stability, and natural non-toxicity, rosemary polyphenol has been widely used in health products and cardiovascular drugs. Rosemary polyphenol can regulate the metabolic and transcriptional changes in HT-29 cells dominated by the production of reactive oxygen species and the coordination of the unfolded protein response signaling pathway under endoplasmic reticulum stress using transcriptomics technology [85]. These findings indicated that transcriptomics is a useful tool to investigate the mechanism of polyphenols in cell protection and cancer chemoprevention.

### 3.3. Proteomics

Proteomics can provide systematic research on the characteristics, quantity, and function of all proteins expressed by a certain organism or cell in the treatment of plant polyphenols. Proteomics technology consists of two-dimensional gel electrophoresis (2-DE), isoelectric focusing, time-of-flight mass spectrometry (TOF-MS), electrospray mass spectrometry (ESI-MS), and capillary electrophoresis mass spectrometry [86]. As an important approach for large-scale study of cellular protein functions, proteomics is often used in revealing molecular mechanisms of tumor pathogenesis and searching for biomarkers. With the rise in proteomics technology, it has gradually become a hotspot by elucidating the anti-tumor mechanism of plant polyphenols in the perspective of protein. For example, the expression of the HSP27 protein related to the growth and apoptosis of the breast cancer cell MCF-7 was regulated by polyphenol resveratrol measured using IFE, SDS-PAGE, and ESI-MS/MS proteomics techniques [87]. The anti-tumor synergistic mechanism of curcumin and irinotecan were also investigated using proteomics approach, identifying 54 differentially expressed protein spots involved in the calcium ion, cellular respiratory chain, and redox pathway in colon cancer [88]. Through SDS-PAGE and LC-MS/MS technology, it was also found that the expressions of histocompatibility antigen and β-2-microglobulin were up-regulated in myeloma cells by gossypol treatment, indicating that gossypol has the function of activating cellular immune response [89]. Through 2-DE and MALDI-TOF/MS analysis, epigallocatechin gallate (EGCG) may activate adenine MAPK by inducing ROS production to inhibit FFA-induced lipid aggregation in human hepatocellular carcinomas (HepG2) cells, leading to inhibiting liver gluconeogenesis [90]. Seventy differential protein expression points were found in HepG2 cells after quercetin treatment through proteomics. Among them, the expression of Ras GTPase-activating-like protein involved in cell migration ability was down-regulated by quercetin, indicating that quercetin can inhibit proliferation and migration of HepG2 cells [91]. In general, proteomics provides a novel approach to explore the anti-tumor mechanism of plant polyphenols in cancer cells, including comparing and identifying the differentially expressed proteins, and clarifying the complex process and molecular mechanism.

### 3.4. Metabolomics

Metabolomics is an approach that analyzes metabolites (<1 kDa) of human and animals after polyphenol treatment, and it is a novel “omics” technique proposed after the emergence of genomics, proteomics, and transcriptomics [92]. Genomics and proteomics tell us what might happen, while metabolomics tells us what had happened with polyphenol treatment. Metabolomics explores the relationship between metabolites and physiological changes by analyzing all metabolites in the organism, and applying liquid chromatograph-mass spectrometry (LC-MS), nuclear magnetic resonance, and gas chromatography (GC)-MS [93]. At present, the most common used analysis method is a combined instrument, such as ultra-performance liquid chromatography to quadrupole time-of-flight-MS (UPLC-Q-TOF-MS) and UPLC-MS/MS.

As a vital direction of foodomics, metabolomics technology was applied to quantify the polyphenols in food, and to target metabolic pathways to explore the molecular mechanism of metabolism (Table 2). Metabolomics has been widely used to study the physiological properties of polyphenols by detecting changes in small metabolites. The effects of green tea polyphenols on human health were explored using LC-MS and GC-MS, which led to the finding that metabolic patterns were changed, including decreased thermogenic carbohydrates as well as increased vitamin synthesis and amino acid metabolism. The variation of gut microflora-related metabolism may be the underlying mechanism of green tea polyphenols in preventing obesity [94]. Based on the metabolomics technology of high-resolution MS, the active polyphenols in litchi decreased serum triglyceride and cholesterol levels caused by a high-fat diet, confirming that litchi polyphenols have the inhibiting activity on cardiovascular disease [95]. In addition, anthocyanins in black soybean seed coats can decrease NO, PGE2, and iNOS and COX-2 expression to exert anti-inflammatory activity through metabolomics in rats [96]. The metabolomics of aging mice under the intervention of EGCG was measured using UPLC-Q-TOF-MS, showing that the metabolic pathway and metabolites was related to the anti-aging effect of EGCG [97]. UPLC-Q-TOF-MS metabolomics was used to study the gastrointestinal defense effect of polyphenol-rich bee pollen, which was found to inhibit the inflammatory response and regulate the metabolites and metabolic pathway in Caco-2 cells [98]. Although the numbers of studies have shown that plant polyphenols have various functions in vivo and vitro, in fact, the bioavailability of plant polyphenols is low [99], and their mechanism that affects health is still not fully clear. In the future, the strategy of enhancing the bioavailability of polyphenols needs to be explored.

### 3.5. Multi-Omics

Omics has a great contribution to the study of the action mechanism of polyphenols. However, the information obtained from the previous reports at a certain level (genome, transcriptome, and proteome) has certain limitations. Therefore, multiple omics should be combined to make correlations from a holistic perspective to systematically elucidate the functions and mechanisms of polyphenols. The regulation of R2R3-MYBs transcription factor is the key to control the synthesis of flavonoids in angiosperms using the combination of metabolome and transcriptome [116,117,118]. Based on multi-omics technology, high-polymer proanthocyanidins was found to reduce the level of metabolites, influence the intestinal microbial community, and have a beneficial effect on metabolic homeostasis [119]. By using 2-DE and MALDI-TOF/MS analysis, EGCG can decrease cell apoptosis and inhibit metastasis of human liver cancer cells [120]. Similarly, it was revealed that resveratrol can regulate the cell cycle of colon cancer cell using SDS-PAGE and LC-MS/MS analysis [121]. In conclusion, multi-omics is a useful approach in the study of the functional activity of plant polyphenols.

## 4. Microbiomics Involved in the Bioactivity of Polyphenols

### 4.1. Regulation of Polyphenols on Gut Microbiota

**Figure 3:**
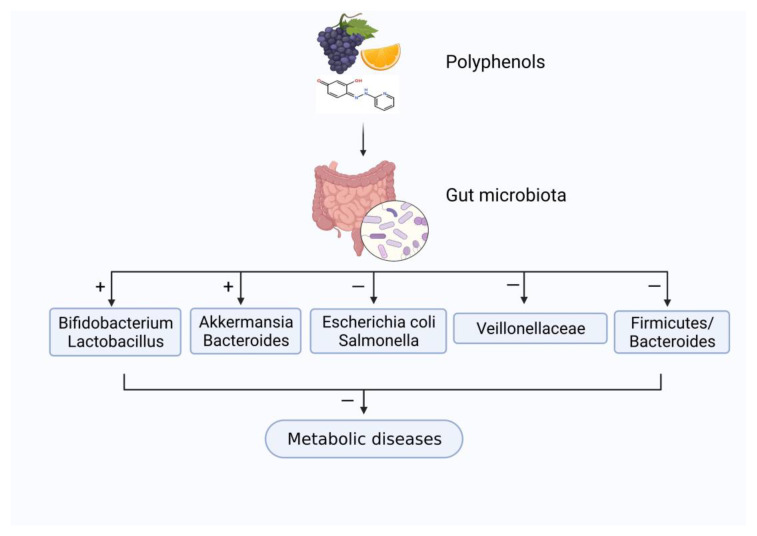
The influence of the interaction between plant polyphenols and gut microbiota on metabolic diseases. “+” means “enhance”; “−” means “weaken”.

Microbiome refers to all microorganisms and genetic information in a specific environment and has beneficial effects in nutrition, metabolism, and immunity [122]. Gut microbiota mainly consists of *Actinobacteria*, *Bacteroidetes*, *Firmicutes*, *Fusobacteria*, *Proteobacteria*, and *Verrucomicrobia*. Among them, *Firmicutes* and *Bacteroides* are the dominant microbiota [123]. Like prebiotics, the polyphenols in diet have received widespread attention for their functional regulatory effects on gut microorganisms, as shown in Figure 3. Polyphenols can inhibit harmful bacteria proliferation (e.g., *Escherichia coli* and *Salmonella*), while promoting the growth of probiotics (e.g., *Bifidobacterium* and *Lactobacillus*).

Intestinal disorders can be inhibited by polyphenols via richening the abundance of beneficial bacteria and microbial diversity. Resveratrol has a promoting effect on *Lactobacillus* and *Bifidobacterium*, which can exert anti-inflammatory effects through reducing pro-inflammatory cytokines and increasing anti-inflammatory cytokines [124,125]. Alpha diversity of gut microbiota was changed, and relative abundances of *Bifidobacterium*, *Feacalibacterium*, *Eubacterium*, and *Coprococcus* were increased by the intake of polyphenols from green tea [126]. The abundance of *Bifidobacteria* and *Lactobacillus* in the gut were increased by the intake of blueberries [127]. In addition, it has been confirmed that inflammatory bowel disease (IBD) is influenced by multiple factors, including the host, microorganisms, and the environment, and the occurrence of IBD is related to gut microbes [128]. In our lab, we found that polyphenol taxifolin changed the composition of colonic microbial community by 16S rDNA sequencing. The change in *Bacteroides*, *Clostridium saccharogumia*, *Clostridium ramosum*, *Sphingobacterium multivorum*, and *Bacteroidetes*/*Firmicutes* ratio caused by dextran sulfate sodium was restored by taxifolin to relieve mice colitis [129]. In conclusion, plant polyphenols can promote beneficial bacteria in the process of regulating intestinal microbes. Once the polyphenols enter the intestinal tract, they will activate the gut microbiota and regulate gut microecology. Conversely, polyphenols can also be used by gut microbiota to produce bioactive molecules (e.g., phenolic acids), which may be the key biologically active effector [130,131], subsequently promoting the health of human and animals.

### 4.2. Combination of Microbiome and Metabolomics in Polyphenol Study

**Figure 4:**
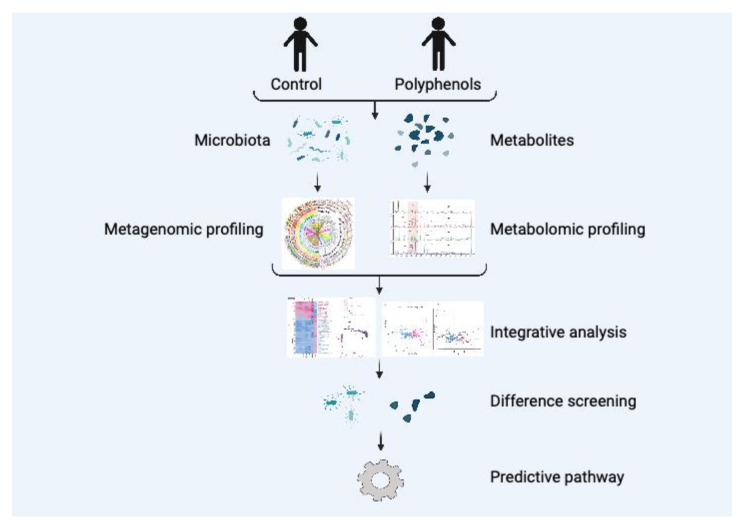
Combining microbiome and metabolomics to investigate the bioactivity of plant polyphenols.

Currently, microbiome technologies mainly contain microbial metagenomics, metametabolomics, macrotranscriptomics, and macroproteomics, allowing us to analyze the microbiome at different levels (e.g., DNA, RNA, protein, and metabolites). At present, numbers of studies on polyphenols mainly focus on metagenomics and metametabolomics. In contrast, their combination to explore the metabolic process of polyphenols is still lacking. The combination of metabolomics and microbiome is a novel approach to explore the specific mechanism of polyphenols. Gut microbiota plays a critical role in health and nutritional status of human and animals [132]. In general, the method of the combination of microbiome and metabolomics is shown in Figure 4. By sequencing the metagenomics of gut microbiota, the corresponding microorganisms can be identified and metabolites can be analyzed using metabolomics technology to discover a novel pathway. In a previous study, primary bile acids were modified into secondary bile acids by clostridium species using 16S amplicon sequencing and metabolomics [133]. It has been verified that polyphenols have various biological activities with a positive influences on gut microbes [134,135,136]. Therefore, the combination of metabolomics and microbiome in the exploration of polyphenols is a trend in the future.

The combination of microbiome and metabolomics has been used to study the effects of plant polyphenols on cardiovascular diseases. Through the metabolomics and genomics analysis of microorganisms in serum, urine, and feces, the risk of cardiovascular disease can be reduced by pomegranate polyphenols [137]. The influence of green tea polyphenols on gut microbiota and micronutrient metabolism was analyzed using metagenomics and metabolomics, and the metabolites of tricarboxylic acid and urea cycle were analyzed using metabolomics and 16S rRNA sequencing, showing that energy conversion was enhanced by green tea polyphenols via promoting the metabolism of gut microbiota in rat [138]. Moreover, the diversity and overall structure of gut microbiota were changed by polyphenols using 16S rRNA sequencing, indicating that polyphenols have an anti-cancer effect. Therefore, it has been speculated that polyphenols can regulate tumor growth by controlling certain bacteria and subsequently changing the cellular components and metabolites [139]. In conclusion, the positive effects of polyphenols on human gut health can be clarified through a microbiome approach. The underlying mechanisms of polyphenols on gut microbiota and metabolites using microbiomics, metabolomics, and multiple omics need to be further explored.

## 5. Conclusions and Further Perspective

In summary, polyphenols are widely distributed in our diet, which mainly come from vegetables and fruits. It has anti-inflammatory, anti-oxidation, anti-tumor, and gut health protection properties. Foodomics (genomics, transcriptomics, proteomics, metabolomics, and multi-omics) emphasized the remarkable efficacy of polyphenols in preventing and treating diseases, especially the anti-inflammatory and anti-tumor activity and gut microbiota regulation. Gut microbes are involved in the metabolic processes of polyphenols. Foodomics and microbiomics have contributed to the study of the mechanism of polyphenols.

With the continuous development of omics technology, integrated analysis technology of omics and multi-omics will help to explore the biological activity and gut microbiota regulation of plant polyphenols. However, the bioactivity, metabolism, and mechanism of polyphenols have not been fully understood. Due to the physical and chemical stability, chelation, food interaction, and gastrointestinal absorption and metabolism, the bioavailability of polyphenols is still low in the body of human and animals. Therefore, it is necessary to combine multiple omics technologies to investigate the biological function mechanism of plant polyphenols in the future. The strategy of enhancing the bioavailability of polyphenols also needs to be further explored. The combined analysis of foodomics and microbiomics is a trend of polyphenol research.

## Figures and Tables

**Table 1:** Classification, sources, and functions of polyphenols.

Polyphenols	Subclass	Sources	Function	Ref.
Phenolic acids		Coffee, berries, kiwi, apple, cherry	Anti-inflammatory, anti-oxidant, antibacterial, antiviral, antiparasitic	[38,39,40]
Stilbenes		Grapes, wine	Anti-inflammatory, anti-oxidant, heart protection, anti-cancer, anti-obesity	[41,42,43]
Lignans		Linseed, sesame, wheat	Anti-tumor, scavenging free radicals, anti-oxidant	[44,45,46]
Flavonoids				
	Isoflavones	Soy, miso	Estrogenic activity, anti-inflammatory, anti-obesity, anti-diabetic, anti-oxidant, cholesterol lowering	[47,48,49]
	Flavones	Parsley, celery, capsicum pepper	Anti-inflammatory, anti-oxidant, regulating glucose and lipid metabolism, anti-virus, anti-bacterial, anti-parasitic	[50,51,52]
	Flavanones	Grapefruit, lemon, oranges	Anti-inflammatory, anti-oxidant, regulating glucose and lipid metabolism, preventing liver steatosis, anti-bacterial, anti-viral, anti-parasitic, anti-fungal	[53,54]
	Flavonols	Berries, onion, broccoli, leek	Anti-inflammatory, anti-oxidant, anti-virus, anti-bacterial	[55,56,57]
	Flavanols	Grapes, cocoa, wine, apricots, green tea, beans	Anti-inflammatory, anti-oxidant, antibacterial, antiviral, antiparasitic, anticancer	[58,59,60]
	Anthocyanins	Berries, black grapes, aubergine, red wine, rhubarb	Anti-inflammatory, anti-bacterial, anti-oxidant, anti-diabetic, anti-cancer, nerve protection, anti-allergic	[61,62,63]
Tannins	Condensed tannins	Cocoa, chocolate, apples, grapes	Anti-oxidant, eliminating free radicals, enhancing immunity, preventing cardiovascular and cerebrovascular diseases, improving hypoxia	[64,65,66]
	Hydrolyzable tannins	Mango, pomegranate	Anti-oxidant, anticancer, phytoestrogens activity	[67,68]

**Table 2:** Metabolomics used in identifying plant polyphenols of food.

Polyphenol-Rich Foods	Polyphenols Identification	MS Based Tools	Ref.
Strawberry	Cyanidin, ellagic acid derivatives, glycosides of quercetin, kaempferol, taxifolin 3-*O*-arabinoside, peonidin, pelargonidin	UHPLC-HR-MS	[100]
Blueberry	Anthocyanins, flavonols, flavan-3-ols, resveratrol, phenolic acids	HPLC-IT-TOF-MS	[101]
Cherry	Hydroxycinnamic acids, anthocyanins, flavonoids	LC-ESI-Q-TOF-MS	[102]
Berry	Delphinidin-3-O-rutinoside-5-O-glucoside; 5-caffeoylquinic acid, quercetin-3-O-rutinoside, quercetin-3-O-glucoside, petunidin-3-O-rutinoside-5-O-glucoside, 3-caffeoylquinic acid, malvidin-3-O-rutinoside-5-O-glucoside, 4-caffeoylquinic acid	UPLC-PDA-Q-TOF-MS	[103]
Grape	Anthocyanins, flavan-3-ols, flavonols, stilbenes	LC-MS	[104]
Apples	Procyanidin, chlorogenic acid, quercetin, (+)-catechin, (−)-epicatechin	UPLC/MS	[105]
Onions	Quercetin-4′-glucoside, quercetin-3,4′-diglucoside	LC-MS/MS	[106]
Cabbage	Quercetin-3-disinapoyl-triglucoside-7-diglucoside, kaempferol 3-di(tri, feruloyldi, sinapoyltri, disinapoyltri)glucoside-7-diglucoside	HPLC-DAD-ESI-MS, HPLC-DAD-MS	[107,108]
Beans	Delphinidin-3-O-glucoside, cyanidin-3-O-glucoside, cyanidin-3-O-sambubioside, pelargonidin-3-O-glucoside, malvidin-3-O-glucoside, cyanidin-3-O-galactoside, petunidin-3-O-glucoside	HPLC-ESI-MS	[109]
Barley	Caffeic acid, catechin, cereals, ellagic acid, ferulic acid, gallic acid, isoscoparin-2″-O-glucoside, p-coumaric acid, procyanidin B2	Q-TOF-LC-MS	[110]
Nut	(+)-catechin, (−)-epicatechin, procatechuic acid, p-hydroxybenzoic acid, quercetin-3-O-rutinoside, naringenin-7-O-glucoside	LC-MS	[111]
Cocoa beans	Flavan-3-ols, procyanidins, (+)-catechin, (−)-epicatechin	HPLC-DAD, HPLC-FL	[112]
Coffee	Phenolic acids, flavonoids, secoiridoids	HPLC-MS/MS	[113]
Green tea	Quercetin-3-O-galactoside, chlorogenic acid, epicatechin, epigallocatechin gallate, proanthocyanidin B2, quercetin-3-O-galactoside	LC-MS	[114]
Wine	Gallic acid, gentisic acid, protocatechuic acid-O-hexoside, protocatechuic acid, caftaric acid, catechin, coumaric-O-hexoside, *p*-hydroxybenzoic acid, caffeic acid	HPLC/ESI-LTQ-Orbitrap-MS	[115]

Note: ESI-LTQ-MS: electrospray ionisation-linear ion trap quadrupole-Orbitrap-mass spectrometry; HPLC-DAD-ESI-MS: high-performance liquid chromatography coupled to photodiode-array detection and electrospray ionization/ion trap mass spectrometry; IT-TOF: ion trap with time-of-flight; Q-TOF-MS: quadrupole-time of flight; UPLC-Q-TOF-MS: ultra-performance liquid chromatography-quadrupole-time of flight-mass spectrometry; UHPLC-HR: UPLC-high-resolution.

## Data Availability

Not applicable.
